# Water expenses by households living in Flanders: Data availability in the Belgian EU-SILC

**DOI:** 10.1016/j.dib.2018.09.011

**Published:** 2018-09-08

**Authors:** Tim Goedemé, Josefine Vanhille

**Affiliations:** Herman Deleeck Centre for Social Policy – University of Antwerp, St. Jacobstraat 2 (M171), 2000 Antwerp, Belgium

**Keywords:** Survey data, Water expenses, European Union, Income, EU-SILC

## Abstract

Few data sources in developed countries contain for a representative sample of households information on water expenses alongside a rich set of reliable information on individual and household characteristics. In this Data in Brief we describe the Belgian EU-SILC data, which we used for ‘Measuring water affordability in developing economies. The added value of a needs-based approach’ (Vanhille et al., 2018) [1]. EU-SILC can be obtained from the Belgian National Statistical Institute and is the most important representative household survey on income and living conditions in the European Union, and contains, among others, an advanced measurement of household income. It is not well-known, though, that national datasets often contain more information, making them suitable for studies that are somewhat outside the ‘core scope’ of EU-SILC. One example is studying the consumption of water by households, as can be done for Belgium. In this article we briefly introduce the Belgian EU-SILC and present the data on water expenses for households living in Flanders. In 2015, 50 per cent less than 23 EUR on water, while 90 per cent spent less than 45 EUR on water.

**Specifications table**

**Table:** 

Subject area	*Economics*
More specific subject area	*Income, living conditions, social policy, environmental management*
Type of data	*Household survey*
How data was acquired	*Face-to-face interviews among representative sample*
Data format	*Raw microdata*
Experimental factors	*Cross-sectional representative surveying is combined with a rotational panel component for longitudinal analysis*
Experimental features	*Focus on income but also covering the domains of demographics, labour, material living conditions, health, education and housing*
Data source location	*Belgium*
Data accessibility	*Data is with this article and available upon request, to be approved by Belgian privacy commission and Statistics Belgium*https://statbel.fgov.be/fr/statistiques/collecte_donnees/enquetes/silc/
Related research article	J. Vanhille, T.Goedemé, T. Penne, L. Van Thielen, B. Storms, Measuring water affordability in developed economies. The added value of a needs-based approach, J Environ Manage (2018) 217: 611–620. DOI: https://doi.org/10.1016/j.jenvman.2018.03.106[1].

**Value of the data**

• Representative sample of Belgian Households – comparable samples and surveys are available for all EU Member States and some non-EU countries, facilitating comparative studies.
• Contains information that is relevant for multidisciplinary studies, as it contains rich background information on individual and household characteristics, with a specific focus on income, material living conditions, demographic characteristics and labour market participation, but also housing characteristics and – in some national datasets – expenditures on utilities.
• The survey has been running since 2003, facilitating for many countries longitudinal analysis.

## 1 Data

EU-SILC is the principal data source in the European Union (EU) for statistics on income and living conditions. It is based on representative household samples in all EU Member States and some non-EU countries. What is less well-known, is that EU-SILC can also be useful for multidisciplinary studies that are slightly out of the ‘core’ of EU-SILC, especially if one makes use of national versions of the data. In [1] we propose a measure of water affordability for developed countries and demonstrate its features on the basis of the EU Statistics on Income and Living Conditions (EU-SILC). We present the EU-SILC microdata and in particular the information it provides on water expenses by households living in Flanders (Belgium).

### 1.1 Target population

The target population consists of persons living in private households. Persons living in institutions (elderly care centres, prisons, hospitals, monasteries, …) are excluded, as well as smaller remote parts of national territories [2].^1^ EU-SILC is organized yearly since 2003.

### 1.2 Scope

EU-SILC consists of detailed microdata on a wide range of variables related to material living conditions. It contains an extensive measurement of personal and household incomes. Apart from that, information is available on demographic characteristics (age, sex, household composition, citizenship status, and education), housing (quality of housing, housing environment, and housing costs), material deprivation, activity status and labour market participation. Thematic modules (e.g. on social participation, housing conditions, over-indebtedness) are also organized. Atkinson, Guio and Marlier [4] provide a recent account of the types of analysis that are possible with EU-SILC.

## 2 Experimental design, materials and methods

### 2.1 Sample design

Samples must be probability samples selected from the target population. Sample sizes and designs vary widely across countries (e.g. [5]). In most countries, samples are selected using a complex design, involving stratification and clustering. Obviously, these characteristics of the sample design have an impact on the sampling variance, which should be taken into account for purposes of statistical inference (for more information, see [6]). Calibrated weights are provided both for the cross-sectional and longitudinal version of the microdata.

### 2.2 Data collection and distribution

Most of the data are collected through personal face-to-face interviews, but several modes of data collection are in use and in some countries many variables (especially related to personal and household income) are extracted from administrative registers, such as tax records and social security records (cf. [7]). The survey is partially ex-ante and mostly ex-post harmonised by making use of a common list of criteria and pre-defined target variables (there is no common template for the questionnaire). This implies that there are important limits to cross-country comparability. Yet, EU-SILC can be considered the main data source for comparative research on income and living conditions across the EU.

National Statistical Institutes are responsible for the data collection, while the statistical office of the European Union, Eurostat, is responsible for bringing the data together and distributing harmonised microdata files (the so-called ‘User Database’, UDB). The data are available free of charge for recognised research institutions.^2^ Eurostat also publishes a wide range of social indicators, based on EU-SILC, on its website. Some countries collect more information than what is available through Eurostat. Therefore, in some cases, as is the case for research into water expenses, it is worthwhile to submit a data request to individual National Statistical Institutes (NSIs), rather than to Eurostat. Access conditions vary between NSIs, though.

### 2.3 Water use in the Belgian EU-SILC

The harmonised User Database distributed by Eurostat contains no separate information on water consumption by households. There is one variable on monthly housing costs which should include expenses on utilities, including water, gas, electricity and heating. For more detailed information on water consumption, national versions of the EU-SILC data can be used. In [1] we make use of the Belgian EU-SILC data of 2015 to work with more detailed information on water consumption separately. In the Belgian microdata, expenses for utilities are recorded at the moment of the survey (early spring 2015). The expenses are surveyed in two steps. First, the respondent is asked whether the household pays (separately) for water. If so, respondents are asked to look up the latest water bill and report the monthly amount.

As tariffs are organised in different ways across the three Belgian Regions, in [1] we restrict our analysis to households living in Flanders, the largest region in Belgium. Not all households report to pay for water while others are unable to indicate their expenditures on water. In total, information on water expenses is missing for about 5.8% of the households in the sample, living in Flanders (Table 1). Partly, this is caused by item non-response, and partly by a reflection of reality, as there is still a share of privately rented accommodations that does not have separate metering.

**Table 1:** Number of respondents in Belgian EU-SILC with information on self-reported monthly water expenses and living in Flanders.

	Households	Individuals
Total number for Belgium	5,943	14,174
Total number for Flanders	3,111	7,464
Number of missing values (Flanders only)	179	331
Total number of observations without missing values (Flanders only)	2,932	7,133

**Fig. 1:**
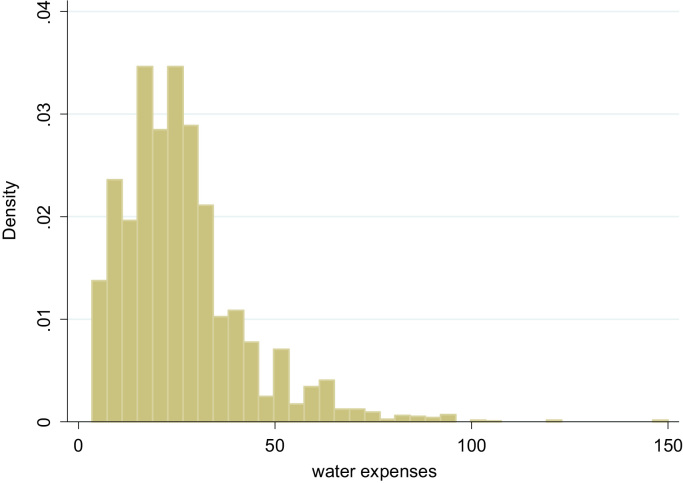
Histogram of water expenses by households living in Flanders, 2015. Source: EU-SILC 2015 (Belgian version).

**Fig. 2:**
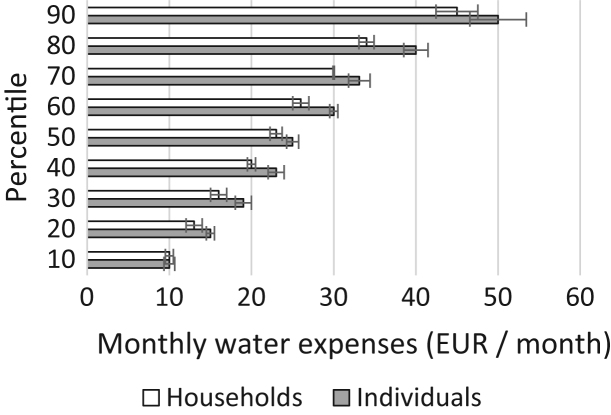
Decile distribution of water expenses, Flanders 2015. Note: 95% Confidence intervals are indicated, taking account of the complexity of the sample design (cf. [6]).

In the following, we show values for those households able to report a monthly expense for water (94% of the sample). Not unsurprisingly, the distribution of water expenses is skewed to the right, as can be observed from Fig. 1. The lowest reported value is 3.4 EUR a month, while the largest is 150 EUR a month. It is not unlikely that the highest reported values are mistakes in the data, but elaborate external validations are not available. Households spent on average around 26 EUR per month (SD = 15.5) on water in 2015. The median household spent about 23 EUR per month on water. The decile distribution can be observed from Fig. 2. About 90% of the households living in Flanders spent in 2015 less than 45 EUR per month on water.

Unfortunately, there is no separate variable on the volume of water consumed. For households reporting water expenses, in principle it is possible to estimate the volume of water consumed by simulating the tariff structure, even though this exercise would come with a margin of error as drinking water tariffs vary between the different area-based water companies and tariffs for wastewater treatment vary between municipalities, while detailed information on the area or municipality of respondents is not available in the microdata.

## Footnotes

1 It is noteworthy that the share of persons living in institutions varies across countries, which may impact upon cross-country comparability (cf. [3]).

2 For a formal description of the data and directions for applying for data access, see http://ec.europa.eu/eurostat/web/microdata/european-union-statistics-on-income-and-living-conditions (last accessed 28 December 2017).
